# Isolation and Molecular Identification of the Native Microflora on *Flammulina velutipes* Fruiting Bodies and Modeling the Growth of Dominant Microbiota (*Lactococcus lactis*)

**DOI:** 10.3389/fmicb.2021.664874

**Published:** 2021-05-21

**Authors:** Qi Wei, Xinyuan Pan, Jie Li, Zhen Jia, Ting Fang, Yuji Jiang

**Affiliations:** ^1^College of Food Science, Fujian Agriculture and Forestry University, Fuzhou, China; ^2^College of Life Science, Ningde Normal University, Ningde, China; ^3^Fujian Higher Education Research Center for Local Biological Resources, Ningde, China; ^4^Fujian Anjoy Food Co., Ltd., Xiamen, China

**Keywords:** *Flammulina velutipes*, microflora, mathematical model, *Lactococcus lactis*, growth

## Abstract

The objectives of this study were to isolate and identify the dominant microorganism in *Flammulina velutipes* fruiting bodies (FVFB) and to develop kinetic models for describing its growth. The native microflora community on FVFB was isolated and identified using morphological examination and high-throughput sequencing analysis. FVFB presented complex microbial communities with dominant microorganisms being *Lactococcus lactis.* Irradiated FVFB were inoculated with the isolated strain of *L. lactis* and cultivated at various temperatures (4, 10, 16, 20, 25, 32, and 37°C). Three primary models, namely the Huang, Baranyi and Roberts, and reparameterized Gompertz models, and three secondary models, namely the Huang square-root, Ratkowsky square-root, and Arrhenius-type models, were developed and evaluated. With the lowest values of mean square error (MSE, 0.023–0.161) and root mean square error (RMSE, 0.152–0.401) values, the reparameterized Gompertz model was more suitable to describe the growth of *L. lactis* on FVFB than both Huang and Baranyi and Roberts models. The Ratkowsky square-root model provided more accurate estimation for the effect of temperature on the specific growth rate of *L. lactis*. The minimum growth temperature predicted by the Ratkowsky square-root model was −7.1°C. The kinetic models developed in this study could be used to evaluate the growth behavior of *L. lactis* on FVFB and estimate the shelf-life of FVFB.

## Introduction

*Flammulina velutipes*, known as golden needle mushroom, is one of the major edible mushrooms employed in factory cultivation in East Asia (Fang et al., 2015). *F. velutipes* had various distinct advantages, owing to its simple cultivation technique, ability of fast fruiting, high yield, desirable taste, and rich in nutrients (essential amino acids, dietary fiber, protein, vitamins, and low in fat). Since *Flammulina velutipes* fruiting bodies (FVFB) have a variety of bioactivities, such as hepatoprotective, antitumor, antihyperlipidemia, immune regulation, and so on (Zhao et al., 2019; Wang and Zhang, 2021), the consumption demands of FVFB were increasing rapidly. The shelf-life of postharvest FVFB is very short (1∼3 days) when stored under ambient condition. The deterioration of postharvest FVFB occurs easily during storage. The main reason might be microbial growth (Shi et al., 2018). Niu et al. (2020) reported that *Pseudomonas* spp. is spoilage organisms on postharvest FVFB. FVFB had a neutral pH value and high moisture content, which could be a suitable medium for the growth of pathogenic and spoilage bacteria.

*Lactococcus lactis* is a Gram-positive bacterium. It is one of the most important microorganisms used extensively in dairy products and other fermented foods (Odamaki et al., 2011). *L. lactis* could survive and grow well in many foods, which could utilize carbohydrates fermentatively and form lactic acid as an end-product. The increasing amount of *L. lactis* and lactic acid in FVFB could significantly reduce its quality by causing softening, browning, rotting, and deterioration. The texture and pH value of FVFB also could be significantly changed with the increasing population of *L. lactis*. Thus, the commercial value of postharvest FVFB could decrease during storage. Based on the beneficial use of *L. lactis* in food fermentation, *L. lactis* has generally been recognized as safe status (Wessels et al., 2004; Food and Drug Administration, 2012). However, some studies had also reported some species of *Lactococcus* to an opportunistic pathogen (Mannion and Rothburn, 1990; Aguirre and Collins, 1993). Rodrigues et al. (2016) suggested that the *Lactococcus* is a potential etiological agent in mastitis outbreak. Zhao et al. (2013) showed that the pathogen causing postharvest water-soaked and sunken lesions on the stipes and decay of *Pleurotus eryngii* was isolated and identified as *L. lactis* subsp. *lactis.* Therefore, *L. lactis* could be potential pathogenic microbes for the mushroom and food industry.

The predictive microbiology models were available to describe the microbial growth, survival, or inactivation in response to environmental conditions (Laurent, 2016; Akkermans et al., 2018). Primary models are used to describe microbial growth data as a function of time under constant environmental conditions (Whiting and Buchanan, 1993). The reparameterized Gompertz model (Zwietering et al., 1990; Buchanan et al., 1997), Huang model (Huang, 2013), Baranyi and Roberts model, and logistic model (Tashiro and Yoshimura, 2019) are generally reported as the primary models. A secondary model deals with the response of parameters appearing in primary modeling approaches as a function of one or more environmental conditions like temperature, pH, etc. (Whiting and Buchanan, 1993). The commonly described secondary models include the Huang square root, Arrhenius type, and Ratkowsky square root (Whiting and Buchanan, 1993; Huang, 2014).

However, there is limited information in the literature about microbial communities and *L. lactis* growth behavior on fresh postharvest FVFB. Therefore, in this study, native microflora on the surface of FVFB were analyzed and discriminated using 16S rRNA gene amplification and high-throughput sequencing. The growth behavior of dominant microflora (*L. lactis*) was studied at different storage temperatures. Mathematical models were fitted to the data and compared to select an accurate model for describing the growth of the dominant microflora. This study could provide useful information to predict the spoilage of FVFB caused by *L. lactis.* The models developed in this study may be used by the food industry and regulatory agencies to estimate the growth of *L. lactis* in FVFB.

## Materials and Methods

### Sample Collection

The FVFB samples were purchased from five different local markets (FV1, FV2, FV3, FV4, and FV5) in Fuzhou, China. FV1 was from Laoguangcun market (farm market), FV2 from Yonghui superstore, FV3 from RT-Mart, FV4 from Walmart, and FV5 from Super Species.

### Native Microflora Isolation and Identification

Twenty-five grams of FVFB were transferred into a sterile plastic bag (19 × 30 cm, Huankai Microbial Co., Ltd., Guangzhou, China) and homogenized in 225 ml 0.1% sterile peptone water (PW). Samples were serially diluted using 0.1% sterile PW. An aliquot of 100 μl was transferred onto Luria–Bertani (LB) agar plates (Huankai Microbial Co., Ltd., Guangzhou, China) and incubated at 37°C for 48 h. Different types of bacterial colonies on LB plates were isolated and transferred into 10 ml of LB broth (Huankai Microbial Co., Ltd., Guangzhou, China). The bacterial cultures were harvested after three consecutive transfers. The cultures were maintained in LB plates and stored at 4°C until use. Each isolated culture obtained was stained by Gram staining and examined using an Eclipse E100 microscope (Nikon, Japan).

Each isolated culture was mixed with 20 μl of double-distilled water and boiled for 2 mins. The bacterial suspension was centrifuged at 12,000 rpm for 1 min. DNA in the supernatants was extracted using a DNA extraction kit (Sangon Biotech, Shanghai, China) according to the manufacturer’s protocol. PCR was carried out on a Mastercycler gradient (Eppendorf, Germany) using 50 μl reaction volumes, containing 2 μl primers (5 μM), 25 μl 2 × Taq PCR Master Mix, 2 μl template DNA, and 21 μl ddH_2_O. The cycling conditions were as follows: initial denaturation at 94°C for 5 min, followed by 35 cycles of denaturation at 95°C for 45 s, annealing at 56°C for 45 s, extension at 72°C for 90 s, and final extension at 72°C for 10 min. The PCR products were qualified and visualized using 1% agarose gel electrophoresis (Sangon Biotech, Shanghai, China) and analyzed by 16S rRNA gene sequencing analysis. Sequences were compared with the National Center for Biotechnology Information (NCBI,^1^) database using the basic local alignment search tool (BLAST) algorithm.

### High-Throughput Sequencing Analysis

The DNA of FVFB was extracted using PowerSoil DNA Isolation Kit (MoBio Laboratories, Carlsbad, CA, United States) according to the protocol provided by the manufacturer. The universal primer sets of 338F (5′-ACTCCTACGGGAGGCAGCAG-3′) and 806R (5′-GGACTACHVGGGTWTCTAAT-3′) were used to amplify the V3--V4 hypervariable region of 16S rRNA genes. High-throughput sequencing was conducted using the Illumina HiSeq 2500 platform at Allwegene Technology Co., Ltd., (Beijing, China). Qualified reads were separated using the sample-specific barcode sequences and trimmed with Illumina Analysis Pipeline Version 2.6. Sequences were analyzed using QIIME (Version 1.8,^2^) software package and in-house Perl scripts to determine alpha diversity and beta diversity. Sequences with the level of 97% similarity were assigned to the same operational taxonomic units (OTUs). The Ribosomal Database Project classifier was used to annotate taxonomic information for each representative sequence.

### Dominant Bacteria and Preparation

*Lactococcus lactis* was obtained from FVFB. The culture of *L. lactis* was revived by inoculating into 10 ml of LB tube and incubating at 37°C for 20 h. After incubation, the bacterial culture was centrifuged at 4,500 rpm for 15 min (4°C), washed three times using 0.1% sterile PW, and then resuspended in 10 ml of 0.1% sterile PW. The suspension was serially diluted to obtain a final concentration of 10^5^ to 10^6^ CFU/ml as working culture.

### Sample Preparation and Inoculation

*Flammulina velutipes* fruiting bodies were purchased from a local market and irradiated (Rice Research Institute, Fujian Academy of Agricultural Sciences, Fuzhou, Fujian, China) at a dose of 5 kg. Afterward, 25 g of FVFB were placed into a sterile plastic bag and inoculated with 0.1 ml of *L. lactis* working culture. The inoculated FVFB samples were incubated at constant temperatures of 4, 10, 16, 20, 25, 32, and 37°C, respectively. Depending on the incubation temperature, samples were retrieved from the incubators at different time intervals. For each sample, 225 ml 0.1% sterile PW was added and homogenized in a stomacher for 20 s (Model BagMixer 400 W, Interscience Co., France). Each sample was serially diluted with PW. Each sample was mixed with 225 ml of 0.1% sterile PW and homogenized in a stomacher (Model BagMixer 400 W, Interscience Co., France). The liquid portion of the homogenized samples was serially diluted with 0.1% sterile PW and plated onto de Man–Rogosa–Sharpe (MRS, Huankai Microbial Co., Ltd., Guangzhou, China) agar plates. All plates were incubated at 37°C for 48 h. The *L. lactis* colonies were enumerated and converted to the logarithm of base 10 or natural base, recorded as log CFU/g or ln CFU/g. Each experiment was repeated three times.

### Primary Models

In this study, three primary models were used for describing *L. lactis* growth: Huang model (Eqs 1, 2, Huang, 2013), Baranyi and Roberts model (Eqs 3, 4, Baranyi and Roberts, 1995), and reparameterized Gompertz model (Eq. 5, Zwietering et al., 1990). The Huang model is expressed as

(1) $$Y\,\left(t\right)=Y_{0}+Y_{\text{max}}-\text{ln}\,\left\{e^{Y_{o}}+\left[e^{Y_{\text{max}}}-e^{Y_{o}}\right]\,e^{-\mu_{\max}\,B\,\left(t\right)}\right\}$$

(2) $$B\,\left(t\right)=t+\frac{1}{\alpha }\,\text{ln}\,\frac{1+e^{-\alpha \,\left(t-\lambda \right)}}{1+e^{\alpha \,\lambda }}$$

where *t* is the time point (h), λ is the lag phase duration (h), *Y*(*t*) represents the natural logarithm of microorganism count (ln CFU/g), *Y*_0_ is the initial microorganisms count (ln CFU/g), *Y*_max_ is the bacterial count (ln CFU/g) at the stationary phase, μ_max_ is the maximum specific growth rate (h^–1^), and α is a constant (α = 4) that defines the transition from the lag phase to the exponential phase of a growth curve (Huang, 2013).

The Baranyi and Roberts model is expressed as

(3) $$Y\,\left(t\right)=Y_{0}+\mu_{\text{max}}\,A\,\left(t\right)-\text{ln}\,\left[1+\frac{e^{\mu_{\text{max}}\,A\,\left(t\right)}-1}{e^{Y_{\text{max}}-Y_{0}}}\right]$$

(4) $$A\,\left(t\right)=t+\frac{1}{\mu_{\text{max}}}\,\text{ln}\,\left(e^{-\mu_{\text{max}}\,t}+e^{-h_{0}}-e^{-\mu_{\text{max}}\,t-h_{0}}\right)$$

where *h*_0_ is the physiological state of the microorganism and equals to λ × μ_max_, and the other variables, i.e., *Y*(*t*), *Y*_0_, *Y*_max_, and μ_max_, are defined as those used in the Huang model. Firstly, the *h*_0_ values are estimated and an *h*_0_ averaged value is established. Then, μ_max_, *Y*_0_, and *Y*_max_ are estimated with fixed *h*_0_ averaged value.

The reparameterized Gompertz model is expressed as

(5) $$Y\,\left(t\right)=Y_{0}+\left(Y_{\text{max}}-Y_{0}\right)\,\text{exp}\,\left\{-e\,x\,p\,\left[\frac{\mu_{\text{max}}\,e}{Y_{\text{max}}-Y_{0}}\,\left(\lambda -t\right)+1\right]\right\}$$

where λ, *t*, *Y*(*t*), *Y*_0_, *Y*_max_, and μ_max_ are identical to those used in the Huang model.

### Secondary Models

Secondary models were fitted to the maximum specific growth rates (μ_max_) estimated from the primary models under different incubation temperatures. The Ratkowsky square-root (Eq. 6, Ratkowsky et al., 1982), Huang square-root (Eq. 7, Huang, 2014), and Arrhenius-type (Eq. 8, Huang et al., 2011) models were used to evaluate the effect of temperature on growth rate.

(6) $$\sqrt{\mu_{\text{max}}}=a\,\left(T-T_{0}\right)$$

(7) $$\sqrt{\mu_{\text{max}}}=a\,{\left(T-T_{m\,i\,n}\right)}^{0.75}$$

(8) $$\mu_{\text{max}}=a\,\left(T+273.15\right)\,\text{exp}\,\left\{-{\left[\frac{\Delta \,G^{\prime }}{R\,\left(T+273.15\right)}\right]}^{n}\right\}$$

In Equations 6–8*a* is a regression coefficient for each model, *T* is the incubation temperature (°C) for microbial growth, *T*_0_ is the nominal minimum temperature (°C), *T*_min_ is the estimated minimum growth temperature (°C), and *a* and *n* are regression coefficients. In Eq. 8, *R* is the universal gas constant [8.314 J/(mol K)], and Δ*G*′ is a type of activation energy related to bacterial growth (J/mol).

### Modeling and Statistical Analysis

Growth curves of *L. lactis* on FVFB at different isothermal temperatures were analyzed using the United States Department of Agriculture (USDA) Integrated Pathogen Modeling Program (IPMP, Huang, 2014). Lag phase durations, specific growth rates, and maximum concentrations of *L. lactis* under different conditions were obtained from IPMP analysis.

## Results and Discussion

### Identification of the Native Microbiota in FVFB

**FIGURE 1:**
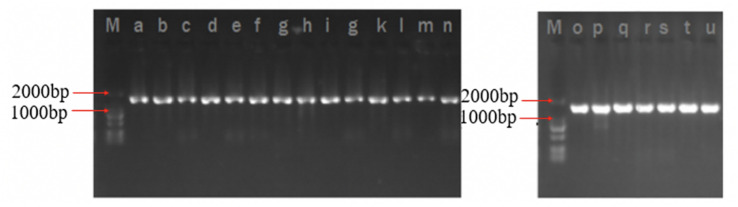
PCR amplification results.

The 16S rRNA gene amplification and high-throughput sequencing analysis were used to identify the native microflora in FVFB. In this research, a total of 21 types of bacterial colonies were isolated and obtained from FVFB in different markets (FV1, FV2, FV3, FV4, and FV5). Each isolated culture was analyzed as shown in Figure 1, and the lengths of DNA fragments extracted from isolated cultures were similar and approximately 1,800 bp. DNA sequences obtained from high-throughput analysis were compared with the NCBI database using BLAST algorithm. A total of 12 species were identified and confirmed (Table 1). FV1 had more species of the bacteria than FV2, FV3, FV4, and FV5, which suggests that FVFB from the farm market (FV1) had a higher risk of being contaminated by the microorganism.

**TABLE 1:** The species of bacteria in *Flammulina velutipes* fruiting bodies.

**Organism**	**FV1**	**FV2**	**FV3**	**FV4**	**FV5**
*Staphylococcus warneri*	+	−	−	−	−
*Ewingella americana*	+	+	+	+	+
*Bacillus amyloliquefaciens*	+	−	−	−	−
*Citrobacter freundii*	+	−	−	−	−
*Raoultella ornithinolytica*	+	−	−	−	−
*Kluyvera cryocrescens*	+	−	−	−	−
*Bacillus paramycoides*	−	+	−	−	+
*Pseudomonas aeruginosa*	−	+	−	−	−
*Lactococcus lactis*	−	+	+	+	−
*Klebsiella aerogenes*	−	−	−	+	−
*Chryseobacterium indologenes*	−	−	−	+	−
*Staphylococcus saprophyticus*	−	−	−	−	+

*+Presence of the corresponding bacterium species based on 16S rRNA sequences.*

*−Absence of bacterium species.*

### Morphological Examination

**FIGURE 2:**
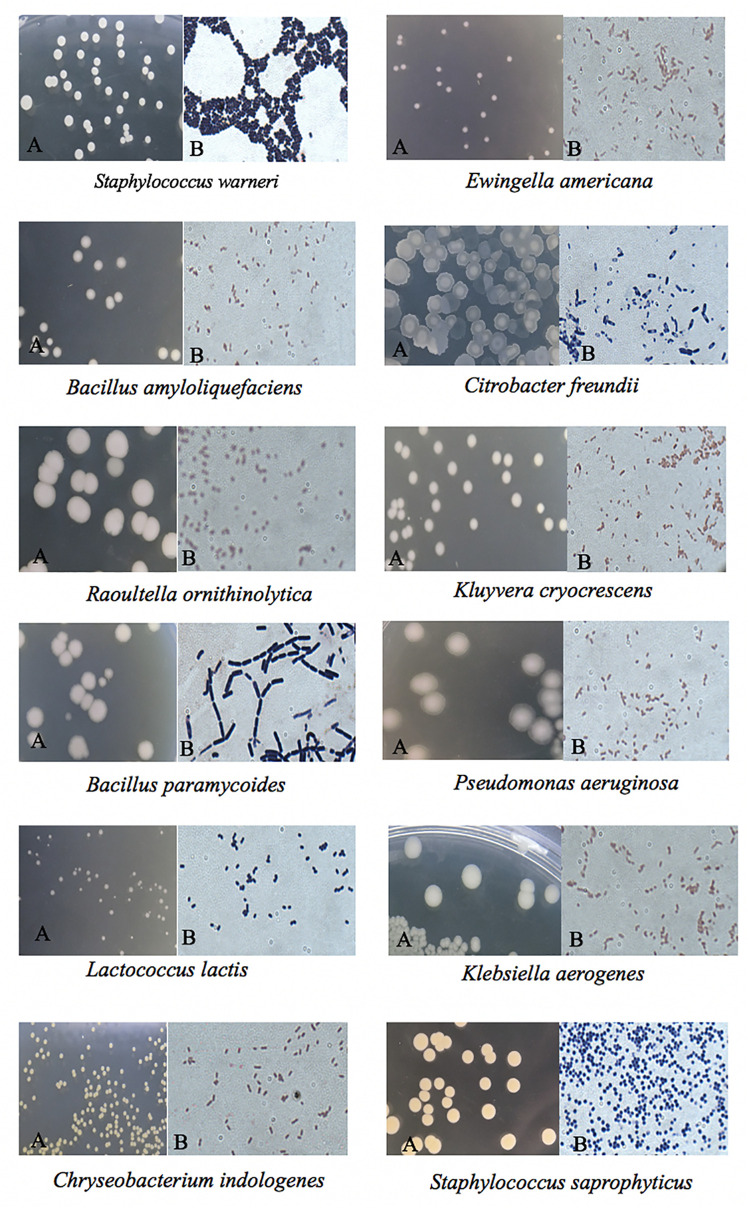
Colony morphology of the identified native microflora in *Flammulina velutipes* fruiting bodies under a microscope: **(A)** without staining and **(B)** Gram staining.

The cultural morphology of each selected bacteria was done and tested with Gram staining and used as a basic separation of bacteria into their taxonomy (Table 2 and Figure 2). A total of five Gram-positive bacteria were obtained, including *Staphylococcus warneri*, *Bacillus amyloliquefaciens*, *Bacillus paramycoides*, *L. lactis*, and *Staphylococcus saprophyticus.* A total of seven Gram-negative bacteria were obtained as follows: *Ewingella americana*, *Citrobacter freundii*, *Raoultella ornithinolytica*, *Kluyvera cryocrescens*, *Pseudomonas aeruginosa*, *Klebsiella aerogenes*, and *Chryseobacterium indologenes*. The morphological examination results demonstrated that the number of Gram-negative bacteria was more than the Gram-positive bacteria isolated in FVFB. The most identified isolates showed the shape of rods except *S. warneri*, *L. lactis*, and *S. saprophyticus.*

**TABLE 2:** Morphological characteristics of identified bacteria in *Flammulina velutipes* fruiting bodies.

**Organism**	**Diameter/mm**	**Form and color**	**Gram reaction**	**Shape**
*Staphylococcus warneri*	0.71∼0.76	Regular, smooth, white	Positive	Cocci
*Ewingella americana*	0.51∼1.79	Regular, smooth, white	Negative	Rods
*Bacillus amyloliquefaciens*	2.27∼4.98	Irregular, smooth, white	Positive	Rods
*Citrobacter freundii*	1.36∼2.70	Regular, smooth, white	Negative	Rods
*Raoultella ornithinolytica*	1.71∼2.63	Regular, smooth, white	Negative	Rods
*Kluyvera cryocrescens*	2.60∼4.21	Regular, smooth, raised, white	Negative	Rods
*Bacillus paramycoides*	2.07∼2.20	Irregular, raised, white	Positive	Rods
*Pseudomonas aeruginosa*	3.38∼5.07	Regular, smooth, flat, pale green	Negative	Rods
*Lactococcus lactis*	1.15∼1.47	Regular, smooth, white	Positive	Cocci
*Klebsiella aerogenes*	1.66∼2.68	Regular, smooth, white	Negative	Rods
*Chryseobacterium indologenes*	0.74∼1.11	Regular, smooth, raised, buff	Negative	Rods
*Staphylococcus saprophyticus*	1.27∼1.60	Regular, smooth, buff	Positive	Cocci

### Native Microflora Composition and Diversity

**FIGURE 3:**
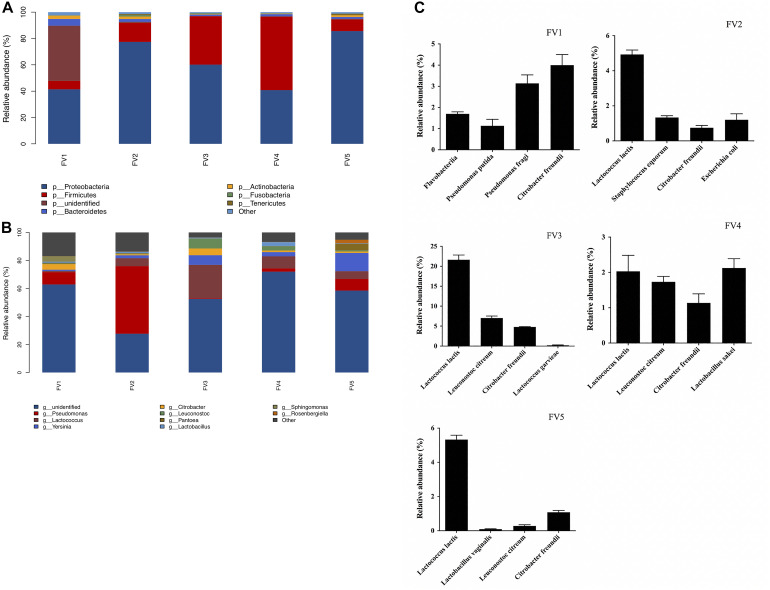
Relative abundance of native microflora in *Flammulina velutipes* fruiting bodies. **(A)** Phylum level, **(B)** genus level, and **(C)** species level.

The bacterial community, relative abundance, and diversity on the surfaces of FVFB were investigated by high-throughput sequencing analysis. The relative abundance at the phylum level of different bacteria in FVFB is shown in Figure 3A. *Proteobacteria*, *Firmicutes*, *Bacteroidetes*, *Actinobacteria*, *Fusobacteria*, and *Tenericutes* were identified on the surface of FVFB. *Proteobacteria* and *Firmicutes* were found to be the dominant microflora in FVFB.

At the genus level, 212 bacteria genera were obtained (the top 10 genera are shown in Figure 3B). Results demonstrated that there were complex microbial communities in FVFB. *Pseudomonas* was determined as the dominant microflora in FV1 and FV2. Based on the observations of Li et al. (2020), the dominant population of microflora was *Pseudomonas* spp., along with small proportions of *Sphingobacterium*, *Pedobacter*, *Corynebacterium*, and *Pasteurella* in *Agaricus bisporus*. Therefore, *Pseudomonas* spp. had high relative abundances in bacterial community structure in mushrooms. *Lactococcus* was determined as the dominant microflora in FV3 and FV4. *Yersinia* was determined as the dominant microflora in FV5. As shown in Figure 3C, the dominant microflora was *Citrobacter freundii* (4%) in FV1 at the species level. However, the dominant microflora was *L. lactis* in FV2 (4.9%), FV3 (21.6%), FV4 (5.3%), and FV5 (2%). These results were the same as those shown in Table 1, which indicated that *L. lactis* was the dominant microflora in FVFB. Lee et al. (2016) demonstrated that lactic acid bacteria were isolated from traditional Korean fermented vegetable. *L. lactis* was one of the most important microorganisms in the dairy industry and generally recognized as safe status. Tormo et al. (2015) reported that *L. lactis* was the dominant species in raw goat milk. However, some studies reported that *L. lactis* was the causal agent of postharvest decay of mushroom (Zhao et al., 2013, 2018). Furthermore, *L. lactis* had a homofermentative metabolism, and it does not directly cause soft rot, but it can produce lactic acid from carbohydrates and create favorable conditions for soft rot symptoms of various vegetables (Baheyeldin and Gahan, 2010; Song et al., 2017). During storage, the increasing amount of *L. lactis* in FVFB could be harmful to its quality by causing softening, browning, sunken lesions, and deterioration in FVFB. Therefore, the growth of *L. lactis* isolated from FVFB was further investigated in this study.

### Mathematical Modeling

*Lactococcus lactis* and other bacteria could grow well in FVFB. At chilled temperature, competition from other psychotropics may not allow *L. lactis* to grow well and reach the maximum population. Due to the limitations of MRS plates, a few other bacteria could also grow in the MRS plate, and *L. lactis* could not be accurately determined by MRS plating (Dave and Shah, 1996). Therefore, to accurately assess the growth of *L. lactis* in FVFB, FVFB was irradiated to eliminate the influence of background microorganisms.

**FIGURE 4:**
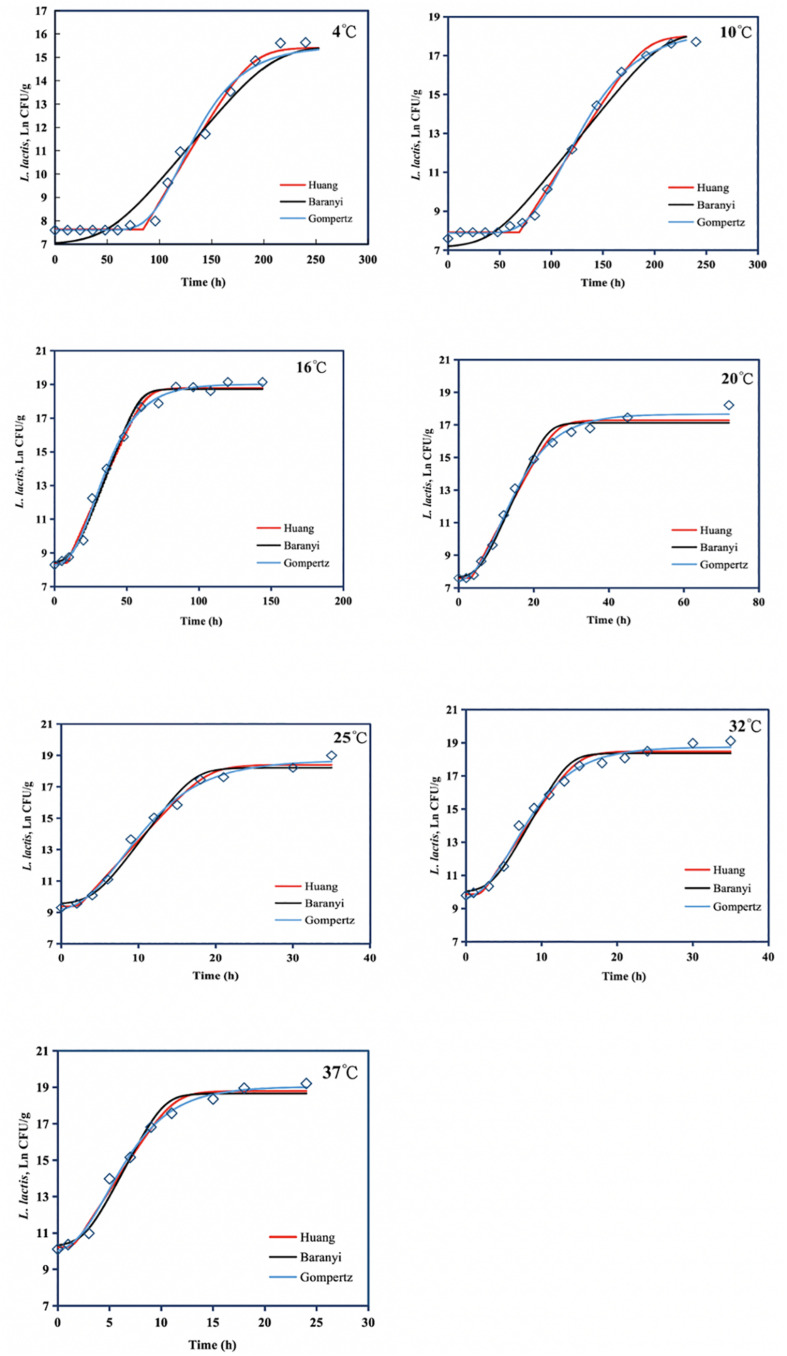
Growth of *Lactococcus lactis* in *Flammulina velutipes* fruiting bodies at temperatures between 4 and 37°C. Solid red line: Huang model, solid black line: Baranyi and Roberts model, solid blue line: reparameterized Gompertz model, diamond: observed growth data.

The Huang, Baranyi, and reparameterized Gompertz models were fitted to the growth data of *L. lactis* in FVFB at different isothermal storage temperatures (4, 10, 16, 20, 25, 32, and 37°C). As shown in Table 3 and Figure 4, the growth curves of *L. lactis* exhibited lag, exponential, and stationary phases at different temperatures. It can be seen that lag time (λ) and specific growth rate (μ_max_) of *L. lactis* were affected by temperature. As temperature increases, the lag time of *L. lactis* decreased, while the growth rates increased. The maximum population of *L. lactis* on FVFB could reach 19.11 ± 0.10 ln CFU/g. For the Huang, Baranyi, and reparameterized Gompertz models, the values of mean square error (MSE) ranged from 0.076 to 0.251, 0.219 to 0.321, and 0.023 to 0.161, respectively, and the root mean square error (RMSE) ranged from 0.276 to 0.501, 0.468 to 0.566, and 0.152 to 0.401, respectively. Compared with the Huang and Baranyi and Robert’s models, the reparameterized Gompertz model had the lowest MSE and RMSE values, indicating that the model was more suitable for describing the growth of *L. lactis* than the Huang and Baranyi and Robert’s models. Wang et al. (2017) demonstrated that the Gompertz model was suitable to estimate the shelf-life of the mushroom *A. bisporus*. Therefore, the Gompertz model was suitable for describing the microbial growth on mushroom.

**TABLE 3:** Estimated parameters for the primary models.

**Temperature**	**4°C**			**10°C**			**16°C**			**20°C**			**25°C**			**32°C**			**37°C**		
**Primary models**	**Huang**	**Baranyi**	**Gompertz**	**Huang**	**Baranyi**	**Gompertz**	**Huang**	**Baranyi**	**Gompertz**	**Huang**	**Baranyi**	**Gompertz**	**Huang**	**Baranyi**	**Gompertz**	**Huang**	**Baranyi**	**Gompertz**	**Huang**	**Baranyi**	**Gompertz**
*Y*_0_ (ln CFU/g)	7.63	7.046	7.604	7.922	7.205	7.878	8.406	8.441	8.211	7.597	7.66	7.281	9.389	9.58	9.048	9.862	10.043	9.482	10.213	10.335	9.905
L95CI	7.356	6.551	7.278	7.681	6.688	7.739	7.699	7.875	7.584	6.809	7.046	6.603	8.491	8.862	8.097	9.066	9.351	8.612	9.333	9.539	8.927
U95CI	7.903	7.541	7.93	8.164	7.722	8.017	9.114	9.006	8.839	8.348	8.273	7.959	10.287	10.299	9.999	10.658	10.736	10.353	11.094	11.132	10.883
*Y*_max_ (ln CFU/g)	15.418	15.481	15.464	18.017	18.189	18.271	18.785	18.718	19.012	17.277	17.128	17.661	18.407	18.217	18.69	18.483	18.369	18.749	18.793	18.66	19.036
L95CI	15.172	15.055	15.153	17.749	17.586	18.098	18.363	18.299	18.627	16.695	16.554	17.206	17.715	17.496	18.049	17.975	17.838	18.282	18.118	17.952	18.407
U95CI	15.663	15.906	15.774	18.286	18.792	18.445	19.207	19.136	19.397	17.86	17.703	18.116	19.099	18.937	19.331	18.991	18.9	19.216	19.467	19.368	19.665
μ_max_ (h^–1^)	0.075	0.054	0.09	0.083	0.066	0.101	0.191	0.221	0.235	0.432	0.519	0.501	0.54	0.665	0.637	0.678	0.818	0.772	0.883	1.085	1.011
L95CI	0.063	0.048	0.07	0.076	0.06	0.095	0.163	0.199	0.199	0.356	0.458	0.432	0.433	0.573	0.522	0.539	0.711	0.634	0.646	0.919	0.779
U95CI	0.087	0.059	0.11	0.09	0.071	0.107	0.22	0.242	0.272	0.508	0.581	0.57	0.648	0.756	0.752	0.817	0.924	0.91	1.12	1.251	1.243
λ (h)	84.427	51.925	89.142	69	42.484	76.285	8.693	12.687	11.358	3.421	5.402	3.85	2.216	4.216	2.475	2.02	3.427	1.949	1.473	2.584	1.527
MSE (ln CFU/g)	0.119	0.319	0.161	0.076	0.321	0.023	0.202	0.219	0.113	0.242	0.284	0.092	0.212	0.303	0.119	0.251	0.318	0.136	0.226	0.302	0.126
RMSE [(ln CFU/g)^2^]	0.345	0.564	0.401	0.276	0.566	0.152	0.449	0.468	0.337	0.492	0.533	0.303	0.46	0.55	0.345	0.501	0.564	0.368	0.476	0.549	0.356

**FIGURE 5:**
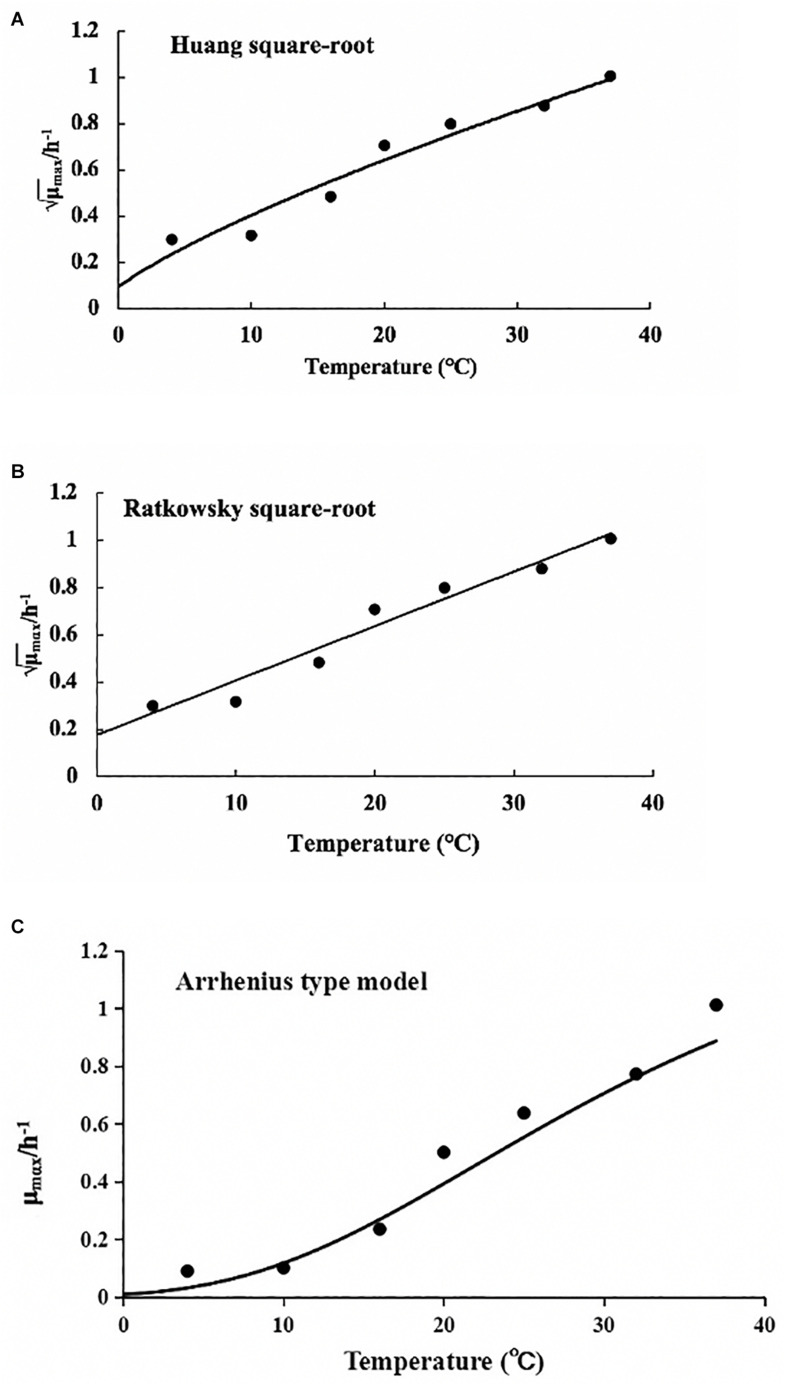
Effect of temperature on the specific growth rate of *Lactococcus lactis* in *Flammulina velutipes* fruiting bodies. **(A)** Huang square-root model, **(B)** Ratkowsky square-root model, and **(C)** Arrhenius-type model.

The values of μ_max_ and λ obtained from the reparameterized Gompertz model were used for secondary models’ development (Figure 5). Table 4 lists the estimated parameters of the Huang, Ratkowsky, and Arrhenius models. The minimum growth temperatures predicted by the Huang square-root model was −1.7°C. The minimum growth temperature estimated by the Ratkowsky square-root model was −7.2°C, which was close to the value (−12°C) reported by Zhou et al. (2014). However, the Arrhenius-type model did not predict minimum growth temperature. Additionally, the values of MSE and RMSE (0.004 and 0.065) for the Ratkowsky square-root model were much lower than those for the Huang square-root model and Arrhenius-type model. Therefore, the Ratkowsky-type model was more accurate to predict the effect of temperature on the specific growth rate of *L. lactis* on FVFB.

**TABLE 4:** Estimated parameters for the secondary models.

**Secondary models**	**Parameters**	**Value**	**Standard deviation**	***t* value**	***p* value**	**MSE [(log CFU/g)^2^]**	**RMSE (log CFU/g)**
Huang square-root model	*A*	0.064	0.005	13.556	3.91E-05	0.005	0.068
	*T*_min_	−1.7	2.142	−0.784	4.69E-01		
Ratkowsky square-root model	*A*	0.023	0.002	10.298	1.485E-04	0.004	0.065
	*T*_0_	−7.2	2.894	−2.48	5.581E-02		
Arrhenius-type model	*A*	0.004	0.001	3.254	3.13E-02	0.005	0.068
	Δ*G*	2,447.797	42.924	57.026	5.662E-07		
	*N*	20.957	8.61	2.434	7.17E-02		

## Conclusion

In this study, morphological examination and high-throughput sequencing were used to investigate the bacterial community in FVFB. *Proteobacteria*, *Firmicutes*, *Bacteroidetes*, *Actinobacteria*, *Fusobacteria*, and *Tenericutes* were the dominant phyla among all the FVFB samples collected from five different markets. *L. lactis* was the dominant microflora in FVFB. FVFB were most likely stored at a refrigerated temperature. However, it might be exposed to temperature abuse during storage. Due to their high moisture and delicate epidermal structure, FVFB were an ideal medium for bacteria growth, including *L. lactis.* The initial count of *L. lactis* in FVFB was estimated at around 9.73 ± 0.41 ln CFU/g. The maximum population of *L. lactis* was 19.11 ± 0.10 ln CFU/g in the stationary phase. This study proved that the Huang, Baranyi, and reparameterized Gompertz models could describe the growth of *L. lactis* in FVFB. The reparameterized Gompertz model was recommended as the primary model for *L. lactis* growth in FVFB, with the lowest MSE and RMSE values, ranging from 0.023 to 0.161, and 0.152 to 0.401, respectively. This study also explored and compared three different secondary models, namely the Ratkowsky square-root model, Huang square-root model, and Arrhenius-type model, and estimated the minimum, optimum, and maximum growth temperatures and the optimum growth rate of *L. lactis* in FVFB. Also, the Ratkowsky square-root model might provide a more accurate estimation of the specific growth rate of *L. lactis* in FVFB.

The effect of temperature on the growth kinetics of *L. lactis* in FVFB was investigated in this study. The primary models and secondary models could be used to predict the growth of *L. lactis* in FVFB during refrigerated storage and temperature abuse. The application of these models can also predict the shelf-life of spoilage microorganism shelf-life of FVFB and be used by regulatory agencies and food processors for conducting risk assessments of *L. lactis* in FVFB. The mathematical models could be used to describe the microbial growth under isothermal conditions in this study. However, temperature abuse during commercial production and distribution, isothermal studies were not suitable to investigate growth kinetics under fluctuating temperature conditions. Therefore, further studies are needed to develop dynamic models under fluctuating temperature conditions during storage.

## Data Availability Statement

The datasets presented in this study can be found in online repositories. The names of the repository/repositories and accession number(s) can be found below: https://www.ncbi.nlm.nih.gov/sra/PRJNA705553.

## Author Contributions

All authors listed have made substantial, direct and intellectual contribution to the work, and approved it for publication.

## Conflict of Interest

XP was employed by company Fujian Anjoy Foods Co., Ltd. The remaining authors declare that the research was conducted in the absence of any commercial or financial relationships that could be construed as a potential conflict of interest.

## Footnotes

1 https://www.ncbi.nlm.nih.gov/

2 http://qiime.org/
